# CT-Guided Stellate Ganglion Pulsed Radiofrequency Stimulation for Facial and Upper Limb Postherpetic Neuralgia

**DOI:** 10.3389/fnins.2019.00170

**Published:** 2019-03-08

**Authors:** Yuanyuan Ding, Peng Yao, Hongxi Li, Zhenkai Han, Shimeng Wang, Tao Hong, Guangyi Zhao

**Affiliations:** ^1^Department of Pain Management, Shengjing Hospital of China Medical University, Shenyang, China; ^2^Department of Anesthesiology, Shengjing Hospital of China Medical University, Shenyang, China

**Keywords:** pulsed radiofrequency, stellate ganglion, facial and upper limb, postherpetic neuralgia, visual analog scale

## Abstract

**Objective:** Postherpetic neuralgia (PHN) is the most common complication of herpes zoster, manifesting as a persistent, spontaneous, knife-like pain or paroxysmal burning that seriously affects a patient’s quality of life. An effective treatment of PHN is lacking. This retrospective study examined the efficacy and safety of stellate ganglion (SG) pulsed radiofrequency (PRF) on facial and upper limb PHN.

**Methods:** Eighty-four patients with PHN on the face or upper limbs were enrolled for the study. Patients were randomly divided into two surgical groups according to the order of enrollment; one group underwent SG block (SG-B group, *n* = 42) and the other underwent SG pulsed radiofrequency (SG-P group, *n* = 42). After surgery, patients were followed at 1 week, 2 weeks, 1 month, 3 months, and 6 months. Observation at each follow-up included basic patient characteristics, visual analog scale (VAS), quality of life (QOL) using Physical Component Summary (PCS), and Mental Component Summary (MCS) to assess, total effective rate, complications and side effects.

**Results:** Compared with preoperative values, VAS decreased in both groups after surgery (*P* < 0.05). In the SG-B group, VAS increased after 1 month, while in the SG-P group, VAS gradually decreased at later follow-up time points. VAS decreased more significantly in the SG-P group after 1 month (*P* < 0.05). PCS and MCS increased in both groups after the operation, and the difference was significant compared with preoperative values (*P* < 0.05). The total effective rates of the SG-B and SG-P groups were 64.3 and 83.3%, respectively. The total effective rate of the SG-P group was higher than that of the SG-B group (*P* < 0.05). The incidence of complications and side effects in the SG-B group was higher than that in the SG-P group (*P* < 0.05).

**Conclusion:** SG pulsed radiofrequency treatment of facial and upper limb PHN is safe and effective. It is a treatment method worth promoting.

## Introduction

Postherpetic neuralgia (PHN) refers to the pain and discomfort that persists for more than 1 month after the disappearance of a herpes zoster (HZ) rash (Fashner and Bell, 2011). PHN is the most common complication of herpes zoster, manifesting as a persistent spontaneous, knife-like pain or paroxysmal burning that seriously affects a patient’s quality of life (Johnson et al., 2010). Herpes zoster in the area of the face and limbs is a high risk factor for PHN due to the sensitivity of the affected area (Forbes et al., 2016). The mechanism of PHN is complex and lacks an effective method of treatment.

The stellate ganglion (SG) is formed by the union of the inferior cervical ganglion with the first thoracic ganglion. The dominant area of the stellate ganglion is the face and upper limbs. Stellate ganglion block (SGB) is an effective, minimally invasive treatment for neurovascular diseases in the dominant area (Jeon, 2016). Blocking the stellate ganglion can effectively improve the blood circulation of the facial and upper limb areas (Kim et al., 2018) and regulate the disordered endocrine system. At the same time, SGB may have preventive effects on PHN by reversing or preventing profound sympathetic stimulation and vasoconstriction, thereby restoring intraneural blood flow, preventing nerve ischemia and damage, alleviating neuralgia and reducing the occurrence of PHN (Boas, 1998; Makharita et al., 2012). However, due to the short duration of local anesthesia, the number of SGB treatments generally need to be increased, which increases the chance of secondary injury. Repeated treatment can lead to patient suffering, poor compliance, and poor quality of life.

Sluijter first proposed pulsed radiofrequency (PRF) for pain treatment (Sluijter, 1997). Since then, it has become a novel means of pain management. PRF delivers short bursts of radiofrequency currents (conducted through a needle) to nervous tissue without damaging the tissue. PRF has a 480 ms pulse intermission period that diffuses the generated temperature so that the temperature of the electrode does not exceed 42°C. This temperature does not cause nerve damage, and thus avoids complications such as hypoesthesia, paresthesia, and dyskinesia. PRF exerts analgesia mainly through neuromodulation. PHN is commonly treated with a combination of therapies.

Postherpetic neuralgia is one of the causes of complex regional pain syndrome (CRPS). While there are a few reports of SG pulsed radiofrequency being used for CRPS (Singh Rana et al., 2015; Kim et al., 2017), there are no reports of SG pulsed radiofrequency being used for PHN in facial and upper limb areas.

In this study, SG pulsed radiofrequency was used to treat facial and upper limb PHN; its clinical efficacy, safety, and long-term quality of life compared with SGB were evaluated.

## Materials and Methods

### Patients

**FIGURE 1:**
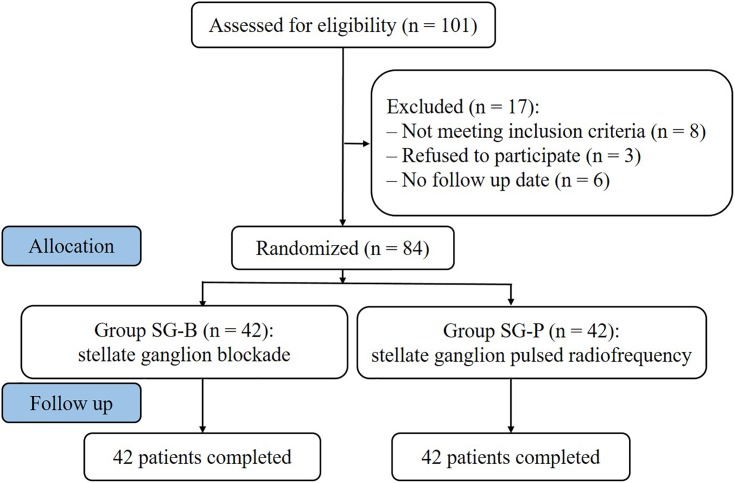
Study flowchart. All 84 patients were included in the treatment.

From January 2015 to December 2016, 84 patients with PHN on the face or upper limbs were enrolled at the Department of Pain Management, Shengjing Hospital of China Medical University (Figure 1). Postherpetic pigmentation or lesions were distributed unilaterally; included were 24 cases of lesions in the area of trigeminal innervation, 18 cases of lesions in the area of facial nerve innervation, and 42 cases of lesions in the upper limbs. Patients were randomly divided into two surgical groups according to the order of enrollment: one group underwent SG block (SG-B group, *n* = 42) and the other group underwent SG pulse radiofrequency (SG-P group, *n* = 42). The study was approved by the Ethics Committee of Shengjing Hospital affiliated with China Medical University. Before surgery, all patients were informed of surgical risks and complications. Written informed consent according to the Declaration of Helsinki was obtained from all patients.

The inclusion criteria were as follows: (1) visual analog scale (VAS) was >5 points within 24 h of enrollment; (2) lesions of the face and upper limbs had healed, but severe intractable pain, local skin hyperalgesia, numbness, and abnormal sensation persisted; (3) the natural course of the disease exceeded 1 month; (4) age >30 years; (5) no nausea, vomiting, dizziness, constipation, or urinary retention before randomization.

The exclusion criteria were as follows: epilepsy, trigeminal neuralgia, intracranial space-occupying lesions, hematological disorders, or abnormal blood coagulation, history of severe liver and kidney dysfunction or history of severe cardiopulmonary disease, pregnancy, and history of drug abuse.

### Surgical Procedure

The patients were placed in a supine position, and the appropriate needle, under CT guidance, was positioned at the base of the C7-T1 parapophysis. A safe route was chosen to avoid injury to the vessel, and the puncture point and puncture angle were clearly defined. After disinfecting the area, a 22G needle or radiofrequency needle was selected. The needle was inserted at the CT positioning angle and gradually advanced under CT guidance until the tip touched the base of C7 and T1 parapophysis, then withdrawn 1–2 mm. No blood or cerebrospinal fluid was drawn back. The SG-B group was injected with 5 mL of 5% lidocaine. The SG-P group was subjected to a radiofrequency test (Baylis Medical Inc., Montreal, Canada): 50 Hz, 0.1–0.3 V sensory test, no neural numbness to the upper limbs or other areas; 2 Hz, 0.4∼1.0 V exercise test, no corresponding segmental muscle tremors and jumping sensation. The PRF was 42°C for 300 s (pulse width 20 ms and frequency 2 Hz). The PRF was performed for two cycles. The needle was withdrawn and pressure applied to the puncture point. No abnormalities were observed. When vital signs were stable, the patient was returned to the ward.

Since antiepileptic drugs are commonly used to treat neuropathic pain, both groups were treated with the antiepileptic drugs carbamazepine (Beijing Novartis Pharmaceutical Co., Ltd., China), gabapentin (Jiangsu Enhua Pharmaceutical Co., Ltd., China), and pregabalin (Pfizer Manufacturing Deutschland GmbH, Germany). The opioid analgesia drug oxycontin (Mundipharma Pharmaceutical Co., Ltd., China), and the neurotrophic drug neurotropin (Nippon Zoki Pharmaceutical Co., Ltd., Japan), were also used. The analgesic effect of all of these drugs were unsatisfactory.

### Observations and Follow Up

Preoperative information included gender, age, pain duration, pain location, affected side of the body, VAS, the dosage of antiepileptic and opioid analgesia drugs.

Patients were followed at 1 week, 2 weeks, 1 month, 3 months, and 6 months with a “blind” method by the non-surgical staff. The following parameters were assessed:

(1) visual analog scale (VAS) to assess the pain level. (0 points – painless, 10 points – unbearable pain).
(2) Thirty six item short-form health survey (SF-36) (Lam et al., 2005) to assess the quality of life (QOL). The questionnaire includes 36 questions; they were used to generate eight scales, including physical, and mental states. The QOL of patients before and after surgery at each time point was assessed. Physical state includes: physical function, physical role, bodily pain, and general health. Mental state includes: vitality, social function, emotional role, and mental health. All the data were summarized to calculate Physical Component Summary (PCS), and Mental Component Summary (MCS).
(3) Total effective rate. The assessment criteria for pain relief is divided into four levels. Subjective symptoms and clinical signs were assessed at 6 months: complete remission of pain (CR, pain relief ≥75%), partial remission of pain (PR, 50%≤ pain relief <75%), mild remission of pain (MR, 25%≤ pain relief <50%), and no remission of pain (NR, pain relief <25%). Significant effective rate (%) = [(CR + PR)/*n*] × 100%, Total effective rate (%) = [(CR + PR + MR)/*n*] × 100%.
(4) Incidence of complications and side effects: including local hematoma, brachial plexus block, pneumothorax, vascular injury (common carotid artery, vertebral artery, vein, etc.), high epidural and subarachnoid block; local anesthetic related adverse reactions (vertigo, dizziness, tinnitus, chills, local anesthetic poisoning, etc.); pain induration and others such as hoarseness/aphonia, pharyngeal foreign body sensation, infection, arrhythmia, etc.

### Statistical Analysis

Data were analyzed using SPSS18.0 statistical software (IBM Corporation, NY, United States). The measurement data were first tested for normality using the single-sample Kolmogorov–Smirnov test. The normal distribution variables were compared using one-way analysis of variance (ANOVA) followed by LSD pairwise comparison; values were expressed as mean ± standard deviation (_$\bar{x}$_ ± SD); the changes of VAS, PCS, and MCS for all time points among the groups were compared using repeated analysis of variance test. The abnormal distribution variables were compared using the Kruskal–Wallis rank sum test; values were expressed as the median±interquartile range. The enumeration data were analyzed by chi square test or Fisher’s exact test. *P* < 0.05 was statistically significant.

## Results

### Patient Characteristics

All patients completed the surgeries, and the procedures were successful. The basic condition of patients in the SG-B and SG-P groups were compared before surgery. There was no significant difference in the gender, age, pain duration, pain location and affected side, VAS, the dosage of antiepileptic and opioid analgesia drugs between the two groups (*P* > 0.05) (Table 1).

**Table 1:** Pre-surgery patient characteristics in SG-B and SG-P groups.

Parameters	Group		*P*-value
	SG-B	SG-P	
Patients (n)	42	42	–
Gender (F/M, %)	20 (47.6%)/22 (52.4%)	19 (45.2%)/23 (54.8%)	0.827
Age (years, range)	55.28 ± 8.35 (34–72)	56.13 ± 8.56 (35–70)	0.569
Pre-surgery pain duration (M, range)	8.32 ± 5.27 (3–17)	8.54 ± 5.42 (3–20)	0.675
Pain location (n, %)			
Trigeminal nerve	20 (47.6%)	21 (50.0%)	–
Facial nerve	6 (14.3%)	7 (16.7%)	–
Brachial plexus	16 (38.1%)	14 (33.3%)	–
Affected side (n, %)			
Right	28 (66.7%)	30 (71.4%)	–
Left	14 (33.3%)	12 (28.6%)	–
Pre-surgery VAS	7.52 ± 1.38	7.61 ± 1.51	0.416
Pre-surgery drug dosage			
Carbamazepine (mg/d, n)	558.65 ± 79.53 (23)	562.02 ± 80.19 (24)	0.493
Gabapentin (g/d, n)	2.78 ± 0.45 (10)	2.81 ± 0.52 (9)	0.715
Pregabalin (mg/d, n)	426.86 ± 73.71 (9)	428.28 ± 74.64 (9)	0.532
Oxycontin (mg/day, n)	43.62 ± 12.78 (42)	44.06 ± 11.95 (42)	0.584


*SG-B, stellate ganglion blockade; SG-P, stellate ganglion pulsed radiofrequency; VAS, visual analog scale. Data are presented as numbers (%) of patients or mean ± SD.*

### VAS Pain Scores

**FIGURE 2:**
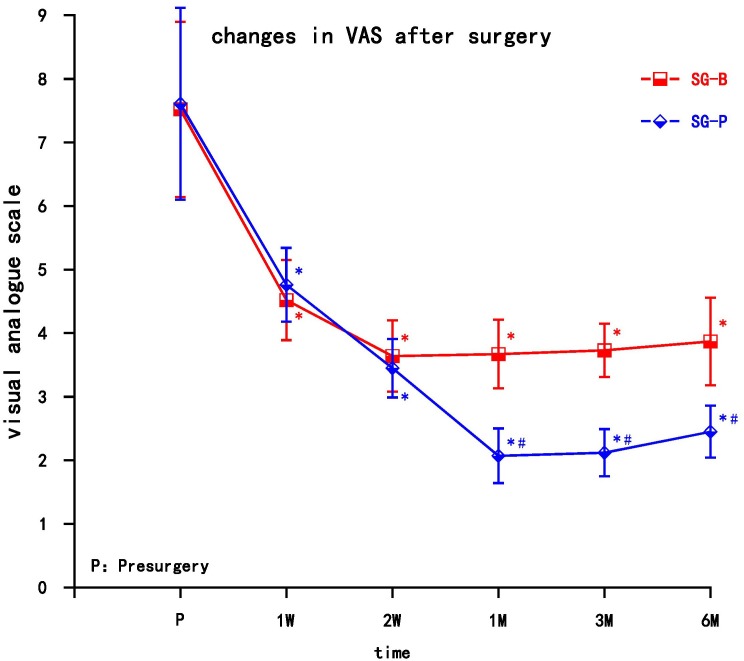
Comparison of VAS pain scores pre-surgery and post-surgery in the two groups. At 1 and 2 weeks, VAS decreased in both groups (*P* > 0.05); VAS decreased significantly in the SG-P group after 1 month (*P* < 0.05). Results are presented as means ± SEMs. ^∗^Compared to pre-surgery, *P* < 0.05; ^#^Compared with SG-B group, *P* < 0.05.

After surgery, VAS decreased in both groups, and the difference was significant compared with preoperative values (*P* < 0.05). At the early follow-up time points (1 and 2 weeks), VAS decreased in both groups, but there was no significant difference between groups. In the SG-B group, VAS increased after 1 month, while in the SG-P group VAS gradually decreased at the later follow-up time points. VAS decreased significantly in the SG-P group after 1 month, and there was a significant difference between the two groups. This difference persisted to the 6 month time point (*P* < 0.05) (Figure 2).

### Quality of Life Evaluation

**FIGURE 3:**
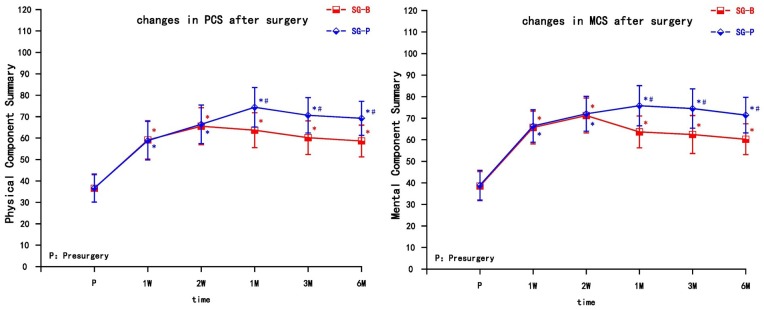
Comparison of quality of life scores (SF-36) pre-surgery and post-surgery in the two groups. At 1 and 2 weeks, PCS and MCS increased in both groups (*P* > 0.05); PCS and MCS increased significantly in the SG-P group after 1 month (*P* < 0.05). PCS, Physical Component Summary; MCS, Mental Component Summary; Results are presented as means ± SEMs. ^∗^Compared to pre-surgery, *P* < 0.05; ^#^Compared with SG-B group, *P* < 0.05.

Both groups of patients achieved varying degrees of improvement in quality of life after pain relief, including physical function, physical role, bodily pain, general health, vitality, social function, emotional role, and mental health. The PCS and MCS increased in the two groups after the operation at each observation time point, and the difference was significant compared with preoperative levels (*P* < 0.05). At the early time points post treatment (1 and 2 weeks), both PCS and MCS gradually increased, but there was no significant difference between the two groups. In the SG-B group, PCS and MCS decreased after 1 month, while in the SG-P group, PCS and MCS continued to gradually increase, indicating a prolonged improvement in the quality of life. PCS and MCS increased significantly after 1 month in SG-P group, and there was a significant difference between the two groups. This difference persisted to the 6 month follow-up time point (*P* < 0.05) (Figure 3).

### Total Effective Rate

At 6 months post-surgery, the total effective rate of the SG-B and SG-P groups was 64.3 and 83.3%, respectively. The total effective rate of SG-P group was higher than that of SG-B group, and the difference was statistically significant (*P* = 0.047) (Table 2).

**Table 2:** Total effective rate in SG-B and SG-P groups (%).

Group	n	Excellent	Effective	Ineffective	The total effective rate(%)
SG-B	42	16	11	15	64.3
SG-P	42	23	12	7	83.3^∗^


*SG-B, stellate ganglion blockade; SG-P, stellate ganglion pulsed radiofrequency; ^∗^Compared with SG-B group, *P* < 0.05.*

### Incidence of Complications and Side Effects

The procedures were completed within 30 min for both groups. Postoperatively, there were no pneumothoraces, no epidural and subarachnoid blockades, no infection, no arrhythmia and no serious complications in either group.

Both groups had local hematoma, nausea and vomiting, hoarseness/aphonia, and pharyngeal foreign body sensation. After local cold compresses, the symptoms gradually resolved within 6 months without subsequent serious adverse reactions. The SG-B group experienced headache, vertigo, and dizziness; complications of brachial plexus block and pain induration also occurred; the total incidence of complications and side effect was 52.4% (22/42). There was no headache, vertigo, or dizziness, and no complication of brachial plexus block or pain induration in the SG-P group; the total incidence of complications and side effect was 16.7% (7/42). The incidence of complications and side effects in the SG-B group was higher than that in the SG-P group (*P* = 0.001) (Table 3).

**Table 3:** Complications and side effects in SG-B and SG-P groups (%).

Complications	Group	
	SG-B	SG-P
Hematoma, n (%)	3 (7.1)	2 (4.8)
Headache/vertigo/dizziness, n (%)	2 (4.8)	0 (0.0)
Nausea/vomiting, n (%)	4 (9.5)	2 (4.8)
Brachial plexus block, n (%)	5 (11.9)	0 (0.0)
Pain induration, n (%)	3 (7.1)	0 (0.0)
Hoarseness/aphonia, n (%)	3 (7.1)	1 (2.4)
Throat foreign body sensation, n (%)	2 (4.8)	2 (4.8)
Incidence of complications and side effect (%)	22 (52.4)	7 (16.7)^∗^


*SG-B, stellate ganglion blockade; SG-P, stellate ganglion pulsed radiofrequency; ^∗^Compared with SG-B group, *P* < 0.05.*

## Discussion

Postherpetic neuralgia is a chronic neuropathic pain. It mainly manifests as spontaneous, allodynia, and hyperalgesia. PHN is associated with the location of herpes and severity of the pain. Patients with herpes zoster in the facial and upper limb areas experience severe pain (Nagasako et al., 2002), and the damaged nerves are more prone to develop PHN. The etiology and mechanism of PHN are unclear, and there are no effective treatments. Because of the special anatomical positions of the facial and upper limb areas, the choice of treatment options is limited. Thus, it is urgent that an effective method of treatment be found.

Postherpetic neuralgia is commonly treated with a variety of drugs, including analgesics, anticonvulsants, antidepressants, and neurotrophic drugs. However, such treatment is generally inadequate for cases involving moderate to severe pain (Sacks, 2013). When herpes invades the area of the face and upper limbs, conventional drug therapy generally needs a longer course of treatment, which can result in a greater chance of adverse reactions. SGB can effectively relieve the acute pain of herpes and reduce the incidence of PHN (Boas, 1998; Wu et al., 2000; Makharita et al., 2012). It is an effective method to treat neurovascular diseases of the face and upper limbs and can effectively relieve the complex regional pain syndromes (CRPS) of the upper body (Datta et al., 2017).

The analgesic mechanism of SGB is still not clear, but most likely involves central and peripheral mechanisms. The central site is mainly located in the hypothalamus (Ranson et al., 1998; Westerhaus and Loewy, 2001), regulating the autonomic nervous system, endocrine system and immune system to maintain the stability of the body’s internal environment (Yokoyama et al., 2000). The peripheral effect of SGB is to block the voltage-gated sodium channels on the nerve cell membrane through local anesthetics, making it difficult for the neural membrane potential to reach the action potential threshold, completely and reversibly blocking the generation and conduction of nerve impulses, blocking the spinal reflex pathway (Lynch and Elgeneidy, 1996; Lipov et al., 2009), and reducing the sympathetic nerve excitability. The functions of the vasculature, glandular secretion, muscle movement, bronchial smooth muscle contraction, and pain transmission controlled by sympathetic nerves are inhibited, thereby dilating blood vessels, increasing the blood flow in the facial and upper limbs, and reducing vascular resistance (Kang et al., 2010; Kim et al., 2018). SGB can improve the neurotrophic status, blocking the vicious cycle of pain (Reinauer et al., 1994). At the same time, it can enhance the defense function and prevent nerve damage (Boas, 1998). SGB can reduce norepinephrine (Mulvaney et al., 2010) and prostaglandins in the brain and plasma, improve ischemic, anoxic and metabolic abnormalities in local tissues, and remove inflammatory mediators by increasing local blood circulation (Park et al., 2010; Gopal et al., 2013; Kim et al., 2013). SGB can also significantly reduce the cortisol, aldosterone, angiotensin-2, 5-hydroxytryptamine and substance P in the blood of patients with pain (Wang et al., 2005; Jeon, 2016), change the lymphocyte subsets and NK cell activity (Yokoyama et al., 2000), inhibit the proinflammatory cytokines IL-1β, IL-6 and TNF-α, regulate the early inflammation responses (Liu et al., 2013), and promote nerve repair. SGB can effectively alleviate facial and upper limb PHN. However, the SG has a special anatomic location and the adjacent structures are complex. Severe complications such as hoarseness, pneumothorax, epidural block, subarachnoid space block, esophageal injury, vascular injury and hematoma formation (Hirota et al., 2017) may occur as a result of the surgery. Anesthetic drugs have a short duration of action. In order to reduce pain and improve efficacy, it is often necessary to increase the number of treatments, which may increase the occurrence of side effects and patient suffering. Therefore, it is desirable to find a therapeutic method that is effective and that can maintain analgesia for an extended period of time.

Radiofrequency is an effective treatment for chronic pain, including conventional radiofrequency (CRF) and PRF. Kastler et al. (2013) reported that CT-guided stellate ganglion CRF treatment of upper limb chronic refractory CRPS-I was more effective than SGB, with an effective rate of 67.6%. CRF produces a high-temperature effect through high-frequency currents, which causes coagulation and degeneration of pain-reducing nerve fibers (Aδ and C-type fibers) and blocks action potentials to achieve analgesia. The mechanism of PRF is different from CRF. The analgesic mechanism of pulsed radiofrequency is unclear (Lin et al., 2014), and it is currently believed that the analgesia is produced by neuromodulation (Cahana et al., 2006; Rehman et al., 2012). The radiofrequency current of PRF is intermittent. This energy transfer does not cause protein coagulation, does not destroy the anatomical basis of pain impulse transmission, and does not cause nerve damage. PRF analgesia is not achieved through temperature effects (Podhajsky et al., 2005; Hamann et al., 2006).

In this study, the decrease in reported pain resulted from a combination of treatments, including analgesic drugs, and neurotrophic drugs. VAS decreased after the operation in both groups, and the difference was significant compared with preoperative values. VAS decreased early in both groups, but VAS in the SG-B group increased after 1 month, while SG-P group still maintained VAS reduction at the later time points. This may be due to the gradual metabolism of local anesthetic drugs over time. As a result, the long-term analgesic substances metabolize and the effect gradually reduces, making it difficult to maintain long-term analgesia. The effect of PRF may be at the microscopic or even subcellular level (Cosman and Cosman, 2005). The effect of neuromodulation is slow, but it can be maintained for an extended period of time.

Inflammation and neurotrophic factors are involved in PHN (Zhao et al., 2017). PRF can regulate the expression of multiple genes in the conduction pathway, enhancing the expression of anti-inflammatory factor genes (GABAB-R1, Na/KATPase, and 5-HT3r) in the dorsal root ganglion, while decreasing the expression of proinflammatory factor genes (TNF-α and IL-6) (Vallejo et al., 2013). PRF has an immunoregulation effect, which has been shown to significantly reduce the level of CD56^+^, CD3^-^, IFN-γ, and NK cell frequency, and increase CD8^+^ T cell frequency and IL-6 in cerebrospinal fluid (Das et al., 2018). PRF has been shown to upregulate the transcription and translation of glial cell line-derived neurotrophic factor (GDNF) in the sciatic nerve and spinal cord (Jia et al., 2016; Hailong et al., 2018) and reduce calcitonin gene-related peptide (CGRP) expression in dorsal root ganglion (Ren et al., 2018). PRF inhibited the excitatory neurotransmitter (glutamate) induced by nociception and then induced an analgesic effect on neuropathic pain (Huang et al., 2016). PRF increased histone acetylation and potassium-chloride cotransporter 2 (KCC2) expression, partially restored GABA synaptic function, alleviated inflammatory pain sensitization (Liu et al., 2017), and attenuated JNK activation in the spinal dorsal horn (Chen et al., 2014). Therefore, PRF effectively relieves pain, and can be maintained over an extended period of time. This long-term analgesic effect avoids repeated treatment of the stellate ganglion. In addition, sensory and exercise nerve tests can be performed before PRF treatment, which will more accurately locate nerves and avoid complications. Therefore, SGB could be used to test the effect first. If the treatment was effective, then SG PRF was used to obtain the positive result. The stellate ganglion is composed of C3–C7 cervical inferior sympathetic ganglia and T1 sympathetic ganglia. The gray traffic branch is connected with the spinal nerves and contains the sympathetic nerve fibers of the brachial plexus. In this study, the SG-B patient group experienced headache, vertigo and dizziness, and there were complications of brachial plexus block and pain induration. No such complications occurred in the SG-P group. The SG-B group had more complications and side effects than SG-P group. The main complications were brachial plexus block and pain induration, which may be related to the diffusion and local injury of local anesthetic drugs. These complications and side effects could affect patient compliance and affected further treatment.

In summary, SG pulsed radiofrequency treatment of facial and upper limb PHN proved superior to SG blockage. The SG pulsed radiofrequency method is safe and effective as it alleviates PHN, improves the quality of life of the patients, and avoids the adverse reaction of local anesthetics. It is a method of treatment worth promoting.

## Author Contributions

TH and PY designed and conducted the study, including patient recruitment, data collection, and data analysis. HL, ZH, and SW collected the data. YD prepared the manuscript draft. GZ analyzed the data. All authors approved the final manuscript.

## Conflict of Interest Statement

The authors declare that the research was conducted in the absence of any commercial or financial relationships that could be construed as a potential conflict of interest.
